# Cholinergic Stimulation of the Adult Zebrafish Brain Induces Phosphorylation of Glycogen Synthase Kinase-3 β and Extracellular Signal-Regulated Kinase in the Telencephalon

**DOI:** 10.3389/fnmol.2019.00091

**Published:** 2019-04-16

**Authors:** Robert A. Mans, Kyle D. Hinton, Cicely H. Payne, Grace E. Powers, Nicole L. Scheuermann, Michael Saint-Jean

**Affiliations:** Department of Biology, Georgia Southern University-Armstrong Campus, Savannah, GA, United States

**Keywords:** acetylcholine receptor (AChR), glycogen synthase kinase (GSK)-3β, extracellular signal-regulate kinase (ERK1/2), telencephalon, cholinergic, zebrafish

## Abstract

The sequencing of the zebrafish genome, the emergence of powerful gene-editing tools, and the development of *in vivo* imaging techniques have propelled the economical zebrafish into prominence as a biomedical research model. Neurodegenerative disorders with a cholinergic component, such as Alzheimer’s and Parkinson’s diseases, are currently modeled using zebrafish. Still, the utility of zebrafish as a research model will not be fully realized until their neurophysiological properties are thoroughly characterized. In mammals, the coupling of cholinergic receptors to the phosphorylation of glycogen synthase kinase-3 β (GSK3β) and extracellular signal-regulated kinase 1/2 (ERK1/2) is of critical importance to cognitive processes and imparts protection against neuropathogenic events. Similarly, it is known that cholinergic receptors are required for learning and memory in zebrafish and that *in vivo* activation of cholinergic receptors induces transient changes in evoked synaptic transmission in the telencephalon. However, the intracellular events mediating cholinergic processes in zebrafish have yet to be elucidated. In the current study, an *ex vivo* drug treatment assay was used to demonstrate that carbachol (CCh)-mediated cholinergic stimulation of the intact adult zebrafish brain induces phosphorylation of GSK3β and ERK1/2 in the zebrafish telencephalon. These findings suggest GSK3β and ERK1/2 may underly cognitive processes in zebrafish.

## Introduction

The contributions of cholinergic neurons span widely across the mammalian brain, including critical roles in cortical and hippocampal processing, attention, decision-making, and the encoding and retrieval of memories (Muir et al., 1992; Bartus, 2000; Rogers and Kesner, 2004; Ferreira-Vieira et al., 2016). Alzheimer’s and Parkinson’s diseases, Down syndrome, amyotrophic lateral sclerosis, and schizophrenia are all marked by cholinergic dysfunction (Yates et al., 1980; Dubois et al., 1983; Tagliavini et al., 1984; Barron et al., 1987; Kato, 1989; Terry, 2008; Ferreira-Vieira et al., 2016; Pepeu and Giovannini, 2017), which may manifest as a profound loss of cholinergic neurons—up to 75% of cholinergic neurons in the basal forebrain, septum and ventral striatum are lost in the AD brain (Davies and Maloney, 1976; Arendt et al., 1983; Nagai et al., 1983; Whitehouse et al., 1983), or as abnormalities in acetylcholine receptor (AChR) function—coupling of AChRs to their downstream signaling proteins is impaired in post-mortem brain tissue from AD patients (Salah-Uddin et al., 2008).

AChRs may be broadly categorized into the G-protein-coupled muscarinic AChRs (mAChRs), which comprise five heterogeneous subtypes (M_1_—M_5_; Caulfield and Birdsall, 1998), and the ionotropic nicotinic AChRs (nAChRs). Frequently, the non-specific cholinergic agonist carbachol (CCh), applied to brain slice preparations and neuronal cell culture has been used to examine the links between AChRs, intracellular protein kinases, synaptic plasticity, and disease pathogenesis. For example, Rosenblum et al. (2000) demonstrated that stimulation of AChRs in rat brain slices and neuronal cultures with CCh induces activation of extracellular signal-regulated kinase (ERK) I/II, which was also shown to be required for induction of long-term potentiation. Additionally, the pairing of AChR-subtype-specific inhibitors with CCh stimulation *in vitro* has been used to demonstrate that cholinergic stimulation induces an M_1_ AChR-dependent long-term synaptic depression in rat hippocampal slices in a mechanism dependent upon ERK I/II activation (Scheiderer et al., 2006, 2008). In the context of neurodegenerative disease, Forlenza et al. (2000) demonstrated that glycogen synthase kinase-3 β (GSK-3β)-mediated phosphorylation of tau is reduced in neurons treated with CCh, and Qiu et al. (2003) showed that CCh treatment is sufficient to reduce amyloidogenic processing of amyloid precursor protein (APP) in hippocampal slices. These and other studies now constitute a large body of evidence demonstrating the importance of the phosphorylated forms of ERK1/2 (Melancon et al., 2013; Giovannini et al., 2015) and GSK3β (Hooper et al., 2007; Serenó et al., 2009) in promoting synaptic plasticity and preventing neuropathology.

While cholinergic neurophysiological processes, and their coupling to intracellular protein kinases, have been extensively elucidated in the mammalian brain, much remains unknown about fundamental neurophysiology in zebrafish (*Danio rerio*), which is gaining in popularity as a model organism to study brain diseases and disorders (Paquet et al., 2010; Xi et al., 2011; Santana et al., 2012; Ng et al., 2013). Anatomically, it is known that functional equivalents of the mammalian amygdala, striatum and hippocampus are represented in the zebrafish brain by the medial zone of the dorsal telencephalon (Lau et al., 2011), dorsal nucleus of the ventral telencephalon (Lau et al., 2011), and lateral zone of the dorsal telencephalon (Rodríguez-Expósito et al., 2017), respectively. Some cellular neurophysiological processes have been shown to be conserved between mammals and zebrafish: as in rodent hippocampus, high-frequency stimulation-induced long-term potentiation of synaptic strength is N-methyl-D-aspartate (NMDA) receptor-dependent in the zebrafish telencephalon (Nam et al., 2004; Ng et al., 2012; Wu et al., 2017). Consistent with these findings, treatment of adult zebrafish with an NMDA-receptor antagonist prevents acquisition of spatial (Cognato et al., 2012) and emotional (Ng et al., 2012) memories. Notably, fear memory impairment in zebrafish caused by treatment with the NMDA-receptor antagonist MK-801 is associated with inactivation of telencephalic ERK (Ng et al., 2012).

Evidence from histochemical (Clemente et al., 2004) and biochemical (Williams and Messer, 2004) experiments indicate widespread cholinergic innervation in the zebrafish brain. Similar to mammals, zebrafish AChRs modulate synaptic strength, as bath application of CCh induces transient synaptic depression in the telencephalon (Park et al., 2008), supporting a role for AChRs in synaptic plasticity in telencephalic circuits. Accordingly, blocking mAChRs using scopolamine prevents spatial and emotional memory function (Kim et al., 2010; Cognato et al., 2012). Additionally, nAChR agonists improve zebrafish spatial discrimination learning (Levin et al., 2006). However, the intracellular signaling events that underlie AChR-dependent cognitive processes in adult zebrafish remain undescribed, and, therefore, further characterization is needed to elucidate the degree of functional conservation between mammalian and zebrafish brains.

Given the importance of GSK3β and ERK1/2 phosphorylation in the neuroprotective and pro-cognitive effects of AChRs in mammals, and the relative paucity of information describing AChR interactions with protein kinases in the zebrafish brain, the current study investigated if CCh-mediated stimulation of the zebrafish brain is sufficient to induce phosphorylation of GSK3β and ERK1/2 in the zebrafish telencephalon.

## Materials and Methods

### Test Subjects

Male and female adult zebrafish (*Danio rerio*) were purchased from a local supplier (Savannah, GA, USA), and maintained as reported by Mans et al. (2018). All experimental procedures were approved by Georgia Southern University Institutional Animal Use and Care Committee (IACUC).

### Brain Extraction

**Figure 1:**
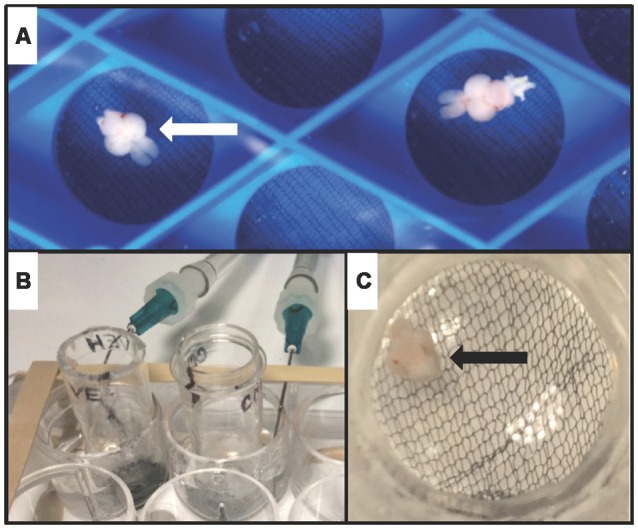
Whole-brain *ex vivo* zebrafish treatment assay. **(A)** Whole brains from adult zebrafish (arrowhead) were removed, and allowed to recover in oxygenated artificial cerebrospinal fluid (aCSF)-filled chambers. **(B)** During treatment, brains were transferred to custom-made treatment chambers inserted into wells of a 12-well tissue culture plate containing vehicle (aCSF) or aCSF plus carbachol (CCh). 95% oxygen and 5% carbon dioxide were delivered to each well *via* a 14G needle. **(C)** View of a single brain (arrowhead) suspended on mesh at the base of a treatment chamber.

Fish were anesthetized by immersion in tricaine methanesulfonate (300 μg/mL) before decapitation, and the head was stabilized in a foam block submerged in ice-cold artificial cerebrospinal fluid (aCSF) infused with 95% oxygen/5% carbon dioxide. aCSF consisted of (in mM): 120 NaCl, 3.5 KCl, 2.0 CaCl, 1.3 MgSO_4_, 1.3 MgCl_2_, 1.25 NaH_2_PO_4_, 26 NaHCO_3_ and 11 glucose (Nam et al., 2004). Extracted brains recovered in oxygenated aCSF at room temperature for at least 30 min prior to use (Figure 1A).

### Drug Preparation

A 10 mM CCh stock solution was prepared in dH_2_O, aliquoted and stored at −20°C before use. On the day of use, 2 μL of CCh stock was diluted in 2 mL of aCSF.

### *Ex vivo* Drug Treatment

Brains were treated in chambers inserted into wells of a 12-well tissue culture plate containing either 2 mL of vehicle (aCSF) or aCSF plus 10 μM CCh (Figures 1B,C) saturated with 95% oxygen/5% carbon dioxide.

### Tissue Collection

After treatment, the telencephalon was removed and immediately placed into 50 μL of homogenization buffer in a 1.5 mL microcentrifuge tube. Homogenization buffer consisted of T-Per Tissue Protein Extraction Reagent (Fisher Scientific) supplemented with protease inhibitor cocktail (Roche) and phosphatase inhibitor cocktail (Pierce). For samples intended for synaptosomal isolation, homogenization buffer volume was increased to 300 μL, and T-Per was replaced with 0.32 M sucrose and 5 mM HEPES (Wirths, 2017). All samples were homogenized at 10,000 rpm for 60 s, then frozen at −20°C.

### Sample Purification

Samples were centrifuged at 1,000 *g* for 10 min at 4°C to remove unhomogenized fragments, and the supernatant was collected for Western blot analysis. To assess synaptic proteins, the crude synaptosomal isolation protocol by Wirths (2017) was adapted. Briefly, homogenate was subjected to 30 pulses with a sonicator and centrifuged at 1,000 *g* for 10 min at 4°C. The supernatant was centrifuged at 12,000 *g* for 10 min at 4°C. The resulting pellet, containing the synaptic fraction, was re-constituted in 50 μL of HEPES-based homogenization buffer.

### Western Blot

Protein concentrations were determined using the Pierce BCA Protein Assay Kit (Thermo Scientific). Samples containing 20 μg of total protein were prepared in SDS sample buffer (Invitrogen, Carlsbad, CA, USA) using standard methods. Samples were resolved by SDS-PAGE in 10% polyacrylamide gels and blotted to PVDF membranes by semi-dry transfer. Membranes were blocked in 3% milk/TBST for 1 h at room temperature prior to application of primary antibody overnight at 4°C. Chemiluminescent protein detection occurred by application of HRP-conjugated secondary antibody for 1 h at room temperature followed by treatment with Clarity Western ECL (Bio-Rad) peroxidase substrate. Blot luminescence was digitally imaged using a ChemiDoc MP Imaging System with Image Lab Software Version 5.1 (Bio-Rad). Protein levels were quantified by densitometry using ImageJ software (NCBI). A mild or harsh stripping protocol (Abcam) was performed prior to blot re-probing.

### Antibodies for Western Blotting

The primary antibodies were rabbit anti-phospho-GSK-3α/β (Ser21/9; Cell Signaling, #9331), rabbit anti-GSK-3β (Cell Signaling, #9315), rabbit anti-tubulin (Tuba1; GeneTex, GTX124965), rabbit anti-phospho-p44/42 MAPK (Erk1/2; Thr202/Tyr204; Cell Signaling, #4370), mouse anti-p44/42 MAPK (Erk1/2; Cell Signaling, #9107), rabbit anti-post-synaptic density-95 (PSD-95; GeneTex, GTX80682), and rabbit anti-beta-actin (Abcam, ab8227). The secondary antibodies were HRP-conjugated anti-rabbit (Cell Signaling, 7074S) or HRP-conjugated anti-mouse (Bio-Rad). All antibodies were diluted in 3% milk/TBST, except phospho-specific antibodies, which were diluted in 2.5% bovine serum albumin/TBST.

### Data Analysis

Data were expressed as mean ± standard error of the mean (SEM). Comparison of data from different treatment groups was performed by Students *t*-test and *P* < 0.05 was considered statistically significant. Data are graphed with control values set at 1.0.

## Results

**Figure 2:**
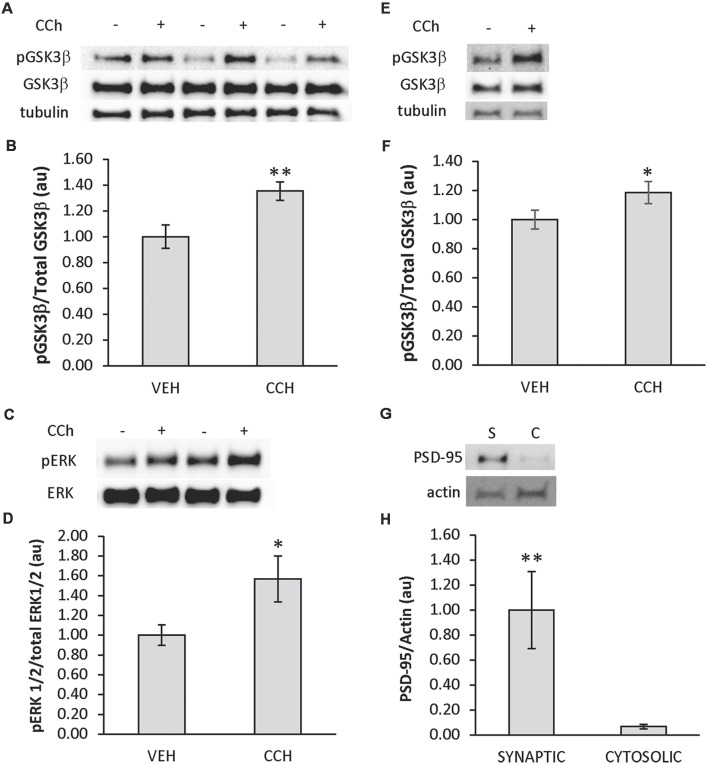
CCh induces phosphorylation of glycogen synthase kinase-3 β (GSK3β) and extracellular signal-regulated kinase 1/2 (ERK 1/2) in the telencephalon of adult zebrafish. **(A)** Representative Western blot of telencephalic tissue from brains incubated in aCSF ± CCh that was probed with pGSK3, GSK3β and tubulin-specific antibodies. **(B)** Quantitative analysis of Western blots showing the ratio of pGSK3β/total GSK3β increased in the telencephalon when CCh was applied *ex vivo* (*n* = 14 veh; 14 CCh). **(C)** Representative Western blot of telencephalic tissue from brains incubated in aCSF ± CCh that was probed with pERK, and ERK-specific antibodies. **(D)** Quantitative analysis of Western blots showing the ratio of pERK/total ERK increased in the telencephalon when CCh was applied *ex vivo* (*n* = 14 veh; 14 CCh). **(E)** Representative Western blots of the synaptic fraction of telencephalic tissue from brains incubated in aCSF ± CCh that was probed with pGSK3, GSK3β, and tubulin-specific antibodies. **(F)** Quantitative analysis of Western blots showing the ratio of pGSK3β/total GSK3β increased in synaptic fractions from telencephalon when CCh was applied *ex vivo* (*n* = 11 veh; 11 CCh). **(G)** Representative Western blot probed with post-synaptic density-95 protein (PSD-95) and actin-specific antibodies confirming PSD-95 in synaptic (S) vs. cytosolic (C) fractions. **(H)** Quantitative analysis of Western blots showing the enrichment of PSD-95 in synaptic fractions (n = 7 synaptic; 7 cytosolic). In plots, values presented as mean ± SE, arbitrary units. Control set at 1.0. *P < 0.05; **P < 0.01 (*t*-test).

In order to test if cholinergic stimulation of the zebrafish brain induces phosphorylation of GSK3β and ERK1/2 in the telencephalon, an *ex vivo* drug incubation assay was developed. The assay consisted of intact adult zebrafish brains placed in custom-made submersion chambers inserted into wells of a 12-well tissue culture plate that were filled with oxygenated aCSF (Figure 1). Using this assay, whole brains isolated from adult zebrafish were treated with the cholinergic agonist CCh (50 μM) or vehicle (veh) *ex vivo* for 10 min followed by biochemical analysis using Western blot. Employing an anti-phospho S9 GSK3β antibody in combination with an anti-GSK3β antibody, we determined CCh treatment induced phosphorylation of GSK3β in the telencephalon (*P* = 0.002; Figures 2A,B), as the mean pGSK3β/GSK3β ratio was 35% higher in CCh-treated samples (*n* = 14) relative to veh-treated controls (*n* = 14). Levels of total GSK3β were also assessed after CCh treatment using the GSK3β/tubulin ratio. Mean total GSK3β was unchanged after CCh treatment (data not shown). To determine if CCh treatment also induced phosphorylation of ERK1/2 in CCh-treated samples, veh- and CCh-treated samples analyzed for GSK3β were analyzed using anti-phospho p44/p42 MAPK and anti-p44/p42MAPK antibodies. We determined that CCh treatment for 10 min caused a 57% increase in the mean ERK1/2 phosphorylation in the telencephalon (*P* = 0.017; Figures 2C,D).

Additionally, due to the importance of GSK3β in synaptic plasticity, CCh treatments were repeated, and the expression of pGSK3β in the synaptic pool of the telencephalon was evaluated in veh-treated (*n* = 11) and CCh-treated (*n* = 11) samples subjected to a crude synaptosomal isolation protocol adapted from Wirths (2017) followed by Western blot analysis. It was determined that CCh induced a 19% increase in the expression of synaptic pGSK3β (*P* = 0.04; Figures 2E,F). In control experiments using remaining samples, the synaptic marker PSD-95 was shown to be enriched in the synaptic samples (*n* = 7) compared to cytosolic samples (*n* = 7; *P* = 0.005; Figures 2G,H). Due to the depletion of samples, a similar analysis of synaptic pERK1/2 could not be performed.

## Discussion

The current study was conducted to further elucidate the degree of functional conservation between mammalian and zebrafish brains. It was determined that widespread stimulation of the cholinergic system *via* bath-application of CCh *ex vivo* results in phosphorylation of GSK3β and ERK1/2 in the telencephalon, a brain area containing functional homologs to the mammalian amygdala, hippocampus and striatum. These results have implications for the study of learning and memory processes in zebrafish, and offer insights into how zebrafish might complement mammalian and tissue-culture model systems in the study of brain diseases with a cholinergic component.

To date, no studies have been published directly testing if zebrafish AChRs—which are required for normal learning and memory in zebrafish (Kim et al., 2010; Cognato et al., 2012)—require GSK3β or ERK1/2 to modulate plasticity. To begin investigating this question, our experiments employed bath application of CCh for 10 min, followed by Western blot analysis of the telencephalon. This treatment has been used previously in mammalian brain slices and neuronal cell culture to demonstrate the involvement of ERK1/2 in hippocampal synaptic plasticity (Rosenblum et al., 2000; Scheiderer et al., 2006). Additionally, an experimental design similar to that of the current study using isolated whole zebrafish telencephalon demonstrated that a 10-min CCh bath application induces synaptic plasticity in the dorsal telencephalic region of zebrafish (Park et al., 2008). Notably, the concentration of CCh used in the current study matches that used in the studies described above. Thus, the CCh-induced phosphorylation of GSK3β and ERK1/2 in the zebrafish telencephalon reported here suggests that zebrafish AChRs may similarly couple to GSK3β and ERK while modulating synapses. It is important to note that control experiments using AChR inhibitors and more isolated experimental systems, such as cell culture, will be required to fully demonstrate zebrafish AChR coupling to GSK3β and ERK1/2, and additional electrophysiology and behavior experiments are required to connect AChRs, intracellular kinases and learning and memory in zebrafish.

With the growth in utilization of zebrafish as a model organism (Paquet et al., 2010; Xi et al., 2011; Santana et al., 2012) has come an appreciation for their value in complementing more traditional mammalian models. For example, it was recently discovered that two parallel molecular signaling pathways may regulate anxiety in humans—one pathway initially characterized in mice and the other subsequently discovered in zebrafish (Xie et al., 2017). The *ex vivo* preparation in the current study offers several advantages. First, the method is highly cost-effective, as the incubation chambers may be constructed from readily-available laboratory supplies, and the volume of drug required for treatments is minimal. Second, the assay employs a brain with largely intact internal circuitry, which increases the likelihood of findings translating to the *in vivo* setting. Conversely, the use of a largely-intact brain presents challenges in predicting the mechanisms underlying the observations made using the *ex vivo* assay. Finally, the assay employs an adult, rather than a larval physiological system, which may be pertinent to studying processes in the aging brain. Therefore, discoveries made in larval systems employing *in vivo* imaging or high-throughput screens could be validated in a fully-developed system using the *ex vivo* assay described here. It will be of great value to continue exploring the potential coupling of zebrafish AChRs to GSK3β and ERK described in the current study, such that the limitations and opportunities implicit to zebrafish as a model organism for complex brain diseases are fully understood.

## Ethics Statement

All experimental procedures were approved by the Georgia Southern University Institutional Animal Use and Care Committee (IACUC).

## Author Contributions

RM conceived and designed the study, performed drug treatments, tissue collection, sample purification and Western blots, completed statistical analysis and interpretation of the data, and prepared the manuscript. KH, CP, GP, NS and MS-J participated in drug treatments, tissue collection, sample purification and Western blots. RM and CP constructed the resting and treatment chambers for extracted zebrafish brains.

## Conflict of Interest Statement

The authors declare that the research was conducted in the absence of any commercial or financial relationships that could be construed as a potential conflict of interest.
